# Influence of Tension Stiffening on the Flexural Stiffness of Reinforced Concrete Circular Sections

**DOI:** 10.3390/ma10060669

**Published:** 2017-06-18

**Authors:** Francesco Morelli, Cosimo Amico, Walter Salvatore, Nunziante Squeglia, Stefano Stacul

**Affiliations:** Department of Civil and Industrial Engineering, University of Pisa, 56126 Pisa PI, Italy; cosimoamico86@gmail.com (C.A.); walter@ing.unipi.it (W.S.); squeglia@ing.unipi.it (N.S.); stefano.stacul@gmail.com (S.S.)

**Keywords:** tension stiffening, circular section, flexural behavior, concrete cracking, numerical model

## Abstract

Within this paper, the assessment of tension stiffening effects on a reinforced concrete element with circular section subjected to axial and bending loads is presented. To this purpose, an enhancement of an analytical model already present within the actual technical literature is proposed. The accuracy of the enhanced method is assessed by comparing the experimental results carried out in past research and the numerical ones obtained by the model. Finally, a parametric study is executed in order to study the influence of axial compressive force on the flexural stiffness of reinforced concrete elements that are characterized by a circular section, comparing the secant stiffness evaluated at yielding and at maximum resistance, considering and not considering the effects of tension stiffness.

## 1. Introduction

Reinforced concrete elements are characterized—even for low force values—by nonlinear behavior, mainly due to the stress–strain relationship of the two forming materials: concrete and steel. The exact modeling of such behavior can prove to be a very hard issue, and for this reason, several simplifications are usually adopted in order to take into account only the most relevant nonlinear aspects affecting the particular studied problem.

**Figure 1:**
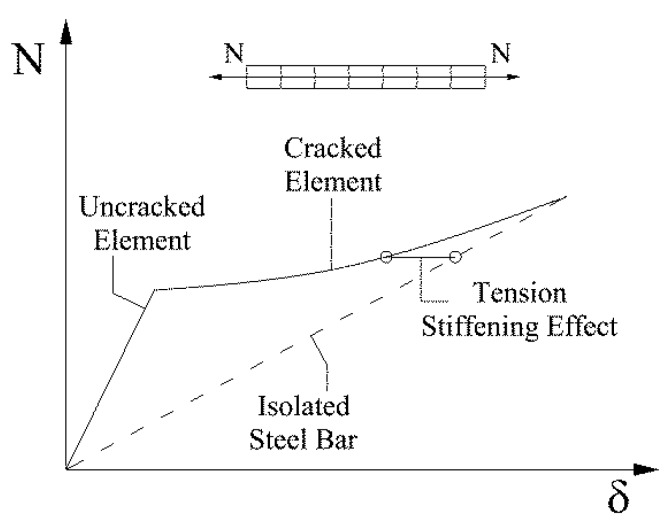
Effects of the tension stiffening on an isolated reinforcing bar.

However, the low tensile strength of the concrete can be indicated as the most influencing nonlinearity source. The idea of the composite material “reinforced concrete” (r.c.) itself was originally developed to overcome such a limitation of the concrete. The low tensile strength does not influence so much the ultimate resistance of a reinforced concrete element. On the contrary, it can modify the element stiffness also for a high value of the external force due to the "tension stiffening". Tension stiffening can be defined as the phenomenon leading to an increase in the stiffness of a concrete section due to the transmission of stresses from the reinforcing bar to the boundary concrete in the tension between two adjacent cracks (Figure 1).

The way of modeling this phenomenon is currently codified by different standards, and several studies have been carried out in the last decade.

Eurocode 2 [1] and CEB-fib Model Code 2010 [2] consider tension stiffening in terms of strain, curvature, or deflection, and interpolate the computed parameter evaluated on the uncracked section and on the fully cracked one using the following expressions.

(1) $$\alpha =\zeta \alpha_{2}+\left(1-\zeta \right)\alpha_{1}$$

(2) $$\zeta =1-\beta \left(\frac{\sigma_{SF}}{\sigma_{S}}\right)$$

where α is the mean value of the parameter of interest (strain, curvature, or deflection) of the element segment comprised between two consecutive cracks; α_1_ and α_2_ are the corresponding values computed in the uncracked and fully cracked sections, respectively; ζ is the distribution coefficient, β is a factor that takes into account long term effects (β = 1.0 for short term effects, β = 0.5 for sustained loads or many cycles of repeated loading); *σ_S_* is the stress in the reinforcement in tension calculated on the cracked section; and σ_SF_ is the evaluated stress under the loading conditions causing first cracking. Eurocode 2 [1] proposes also an expression for the evaluation of the crack’s interaxis mean value, s_rm_:

(3) $$s_{rm}=50+0.25K_{1}K_{2}\frac{\Phi_{s}}{\rho_{p,eff}}\left[\text{in mm}\right]$$

where:

− Φ_s_ is the mean value of the reinforcing bars’ diameter;
− K_1_ is a coefficient that takes into account the bond properties of bond reinforcement;
− K_2_ is a coefficient that takes into account the distribution of strain (pure tension or bending); and
− ρ_p,eff_ = A_st_/A_ct_ is the effective reinforcement ratio evaluated as the ratio between the area of the reinforcing bars, A_st_, contained within the concrete cross section portion effectively influenced by the bars in the formation of cracks.

The effects of tension stiffening on the flexural behavior of r.c. elements are considered in many other studies. The first ones can be attributed to Vecchio and Collins [3], Collins and Mitchell [4], and Belarbi and Hsu [5,6]. The results of such studies are compared, and their differences well explained by the research of Bentz [7], which proposes also the use of an expression that takes into account the different bond conditions, and leads to a better estimation of the crack’s width and stiffness at service load of r.c. elements. Salvatore et al. [8] studied its effect on flexural behavior, with particular attention to a ductility evaluation of rectangular concrete sections reinforced with special, dual-phase [9], steel bars. Shukri et al. [10] studied the tension stiffening contribution of near-surface mounted Carbon Fiber Reinforced Polymer (CFRP) to the behavior of strengthened r.c. beams. Shukri et al. [11] introduced tension stiffening effects to develop a mechanical model for the simulation of r.c. hinges under reversed cyclic loading. Sato et al. [12] extended the results to the r.c. members with externally bonded fiber-reinforced polymers, providing models to estimate crack spacing and the influence of tension stiffening effects. Stramandinoli et al. [13] developed a model in which the tensile stress–strain curve of concrete displays an exponential decay in the post-cracking range, defined by a parameter that depends on the reinforcement ratio and on the steel-to-concrete modular ratio. The numerical results obtained by the model showed good agreement with several experimental results on simply supported beams with rectangular cross sections tested under 4-point bending. Lee et al. [14] presented a tension stiffening model able to calculate average tensile stresses in concrete after the yielding of reinforcement in the r.c. elements has been subjected to uniaxial tension, shear, or flexure. Kaklauskas et al. [15] studied the effects of shrinkage on tension stiffening on rectangular sections with symmetrical or asymmetrical reinforcement, providing free-of-shrinkage tension stiffening relationships. Soltani et al. [16] developed a computational model for the analysis of r.c. membrane elements that have been subjected to general inplane stresses, considering the effects of tension stiffening, the stress transfer across cracks due to aggregate interlock, and dowel action with consideration to the kinking effect of reinforcements at the crack plane. The comparison of the method through a comparison with some experimental results demonstrated the accuracy of the proposed model.

An important aspect to consider in the study of the tension stiffening phenomenon is the definition of the "effective area", defined as the portion of concrete surrounding the reinforcing bar involved in the transmission of stresses from the bar to the concrete itself.

**Figure 2:**
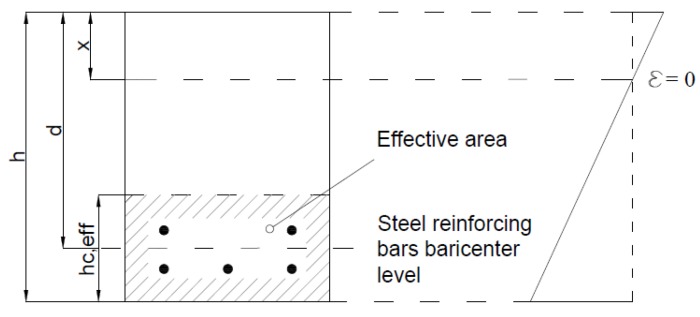
Effective area for rectangular section defined by Eurocode 2 [1].

Eurocode 2 [1] defines the effective area, only for typical rectangular sections, as the area having the same width of the section and a height h_c,eff_ equal to the minimum among 2.5(h − d), (h − x)/3 and h/2, where the meaning of the symbols is well explained by Figure 2. CEB-fib Model Code 2010 [2] suggests similar values, stating that, in the absence of a more refined model, the effective concrete area in tension can be assessed as 2.5(h − d) < (h − x)/3.

Several authors have proposed different expressions for the evaluation of the effective area. Manfredi and Pecce [17] recommended a refined fiber model for the analysis of r.c. beams, which includes an explicit formulation of the bond–slip relationship that employs an effective area around the reinforcement that occupies the whole width of the section and has a height h_c,eff_ = (c + 8.5ϕ), with c being the concrete cover and ϕ the reinforcing bar diameter. Braga et al. [18] studied a slip model that also takes into account hardening phenomena. Kwak and Song [19], in their study on an analytical model which can simulate the post-cracking behavior and tension stiffening effect in a r.c. tension member, proposed that the effective area of concrete in tension can be represented by A_c,eff_ ≈ 1/4(1 + nρ)bh, with b and h the width and height of the section, respectively, n = Es/Ec, and ρ the ratio of steel reinforcement (As/bh). Castel et al. [20] came up with the following expression for A_c,eff_, which is based on a multi-linear stress profile in the full depth of the concrete section between the flexural cracks:

(4) $$A_{ct,eff}=\frac{b\left(d-x-a/2\right)}{2}+b\cdot a+b\left(h-d-a/2\right)\left[1-\frac{b\left(h-d-a/2\right)}{2\left(d-x-a/2\right)}\right]-A_{s}$$

**Figure 3:**
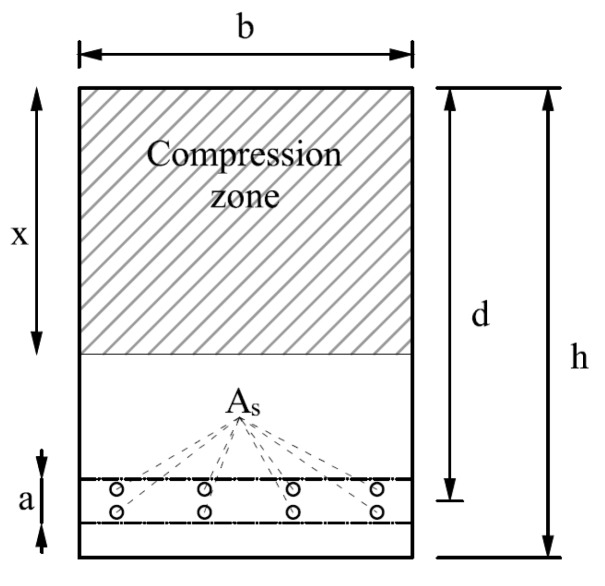
Geometrical parameters used in Equation (4).

The meaning of the symbols is explained in Figure 3. 

All of these works deal with the definition of the effective area on rectangular r.c. sections. Very little work has been done on circular sections, even if they represent important elements in the field of r.c. construction. It is in fact sufficient to think about the bridge piers or pile foundations that are realized with circular sections: bridge piers are often realized this way, while pile foundations practically always are. Wiese et al. supplied an expression for the determination of the effective area of symmetrically reinforced circular sections, idealizing the reinforcement as a continuous ring. J. F. Carbonell-Marquez et al. [21] presented a definition of the effective area in circular cross sections for both symmetric and asymmetric layouts, and demonstrated the validity of the proposed expression by comparing it with the experimental results on r.c. members subjected to pure flexure. Mondal and Prakash [22] came up with an improved analytical model for r.c. circular columns under combined axial-torsional load conditions, demonstrating that neglecting tension stiffening can lower sensibly the accuracy of the numerical/analytical models used in predicting test data.

The influence of tension stiffening on the global behavior of elements with a circular cross section and subjected to the combined axial–flexural action has not, however, as far as the authors know, been ever quantified. The question of if, or in which conditions, it is necessary to model tension stiffening still remains without clear answers. Moreover, the influence on tension stiffening effects of the axial force, to which circular cross section elements such as bridge piers and pile foundations are usually subjected, or of the reinforcement ratio, has never been quantified.

Within the present paper, the modeling approach adopted by Salvatore et al. [8] for rectangular sections is enhanced and adapted to circular, symmetrically reinforced, cross sections. The reliability of the model’s results is then tested, comparing them with the experimental results of tests carried out on circular elements characterized by different reinforcement ratios, and subjected to a combined axial force and bending moment. Finally, a parametric analysis using the proposed model is executed, to estimate the influence of various parameters (axial force, reinforcement ratio) on the global behavior of r.c. elements having a circular cross section.

## 2. Modeling Tension Stiffening

**Figure 4:**
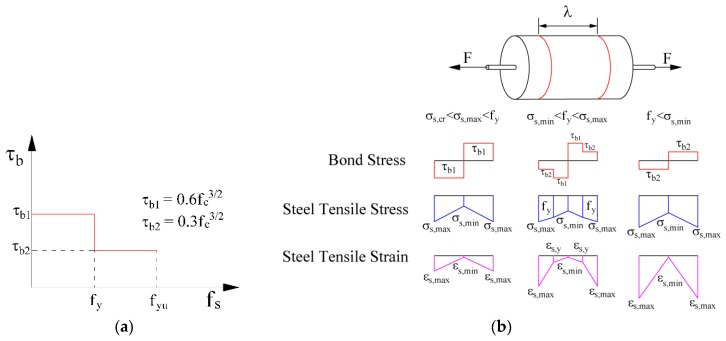
(**a**) Bond–slip relation for the steel–concrete interface; (**b**) adherence, stress, and strain in the steel.

Salvatore et al. [8] proposed a model based on the CEB-fib Model Code 1998 [23] approach, upholding the classical hypotheses of plane cross-sections and perfect adherence between steel bars and the surrounding concrete, even after cracking, in all sections (cracked and uncracked ones). The bond–slip relation is assumed to be rigid–plastic, as illustrated in Figure 4a, where τ_b1_ is the bond stress in the elastic phase, and τ_b2_ is the bond stress at yielding. The bond and steel tensile stresses, together with the steel tensile strain resulting from the application of an increasing force to the steel bar, are schematically shown in Figure 4b.

To consider the deformational effects consequent to sliding between the steel and the concrete and the consequent redistribution of stresses, a fictitious elasticity modulus, E_ct_, for the concrete in tension in the post-cracking phase is defined.

The definition of E_ct_ is derived from the equilibrium equation of an infinitesimal length of bar surrounded by the portion of concrete involved in the transmission of stresses from the bar to the concrete itself. On the base of the stress condition of the steel bar, Salvatore et al. [8] derived the following expression of E_ct_:

for σ_scr_ ≤ σ_s,max_ < f_y_:

(5) $$E_{ct}(x)=\frac{4}{\Phi_{s}}\frac{A_{s}}{A_{c}}\tau_{b1}x\frac{1}{\frac{\sigma_{s,\max}}{E_{s}}-\frac{4}{\Phi_{s}E_{s}}\tau_{b1}x}$$

for σ_s,min_ ≤ f_y_ < σ_s,max_ and x ≥ x_y_:

(6) $$E_{ct}(x)=\frac{\left(\frac{4}{\Phi_{s}}\tau_{b1}x-\frac{4}{\Phi_{s}}\left(\tau_{b1}+\tau_{b2}\right)x_{y}\right)A_{s}}{\left(\frac{\sigma_{s,\max}}{E_{s}}-\frac{4}{\Phi_{s}E_{s}}\left(\tau_{b1}+\tau_{b2}\right)x_{y}+\frac{4}{\Phi_{s}}\tau_{b1}x\right)A_{c}}$$

for f_y_ < σ_s,min_ or σ_s,min_ ≤ f_y_ < σ_s,max_ and x < x_y_:

(7) $$E_{ct}(x)=\frac{\frac{4}{\Phi_{s}}\frac{A_{s}}{A_{c}}\tau_{b2}x}{\varepsilon_{y}+\frac{\sigma_{s,\max}\left(\varepsilon_{u}-\varepsilon_{y}\right)}{f_{u}-f_{y}}-\frac{4\left(\varepsilon_{u}-\varepsilon_{y}\right)}{\Phi_{s}\left(f_{u}-f_{y}\right)}\tau_{b2}x-f_{y}\frac{\left(\varepsilon_{u}-\varepsilon_{y}\right)}{\left(f_{u}-f_{y}\right)}}$$

where σ_s,min_ and σ_s,max_ are the minimum and maximum stresses in the bar occurring respectively in the midline section and in the cross-sections of the element where the crack forms; σ_scr_ is the stress in the steel upon first cracking; and, finally, x_y_ is the distance from the cracked section where the stress in the steel begins to be lower than the yield stress.

### Observations

The modelling approach adopted by Salvatore et al. [8] briefly described in the previous paragraph suffers, however, from some inaccuracies. In the case of the reinforcing bar subjected to stresses lower than the yield ones, the elasticity modulus in tension E_ct_ would be defined by Equation (5), but the following observations can be made:

− for $\frac{\sigma_{s,max}}{E_{s}}-\frac{4}{\Phi_{s}E_{s}}\tau_{b1}x=0$, $E_{ct}\left(x\right)\to \infty$. This result is unrealistic given that the upper limit of E_ct_(x), E_ct,lim_ should be at least equal to the elastic modulus of the concrete in compression, E_c_.
− for $\frac{\sigma_{s,max}}{E_{s}}-\frac{4}{\Phi_{s}E_{s}}\tau_{b1}x<0,\;E_{ct}\left(x\right)<0$. This result has no physical meaning, too. The lower limit of E_ct_ should be equal to zero (corresponding to the cracked section).

**Figure 5:**
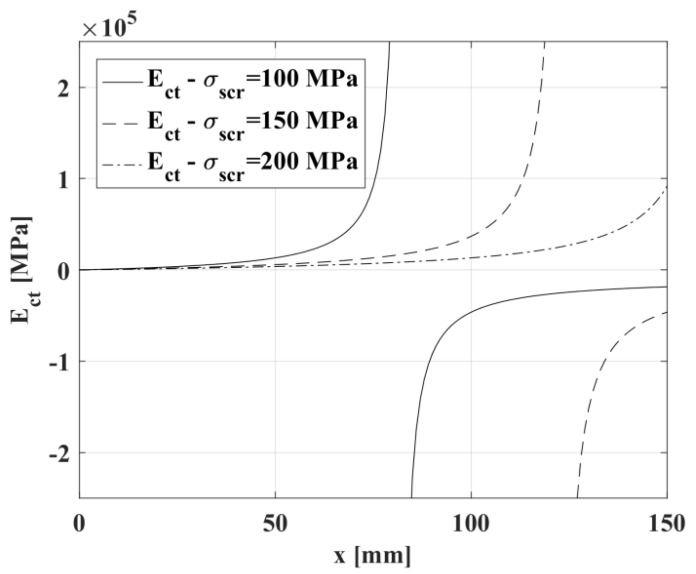
Elasticity modulus in tension, E_ct_, as a function of the distance from the cracked section, x, for different values of the stress in the steel upon first cracking, σ_scr_.

In Figure 5, Equation (5) is plotted for different values of σ_scr_.

It is immediate to understand that, in order to obtain real physical meaning, the fictitious elasticity modulus in tension E_ct_ should respect the following limits:

(8) $$0\leq E_{ct}(x)<E_{ct,\lim}$$

where E_ct,lim_ represents the likely maximum value of the elasticity modulus in tension, assumed, in the present study, to be equal to the concrete elasticity modulus in compression.

For this reason, we assumed, for σ_scr_ ≤ σ_s,max_ < f_y_,:

(9) $$E_{ct}(x)=\{\begin{array}{ccc} \frac{4}{\Phi_{s}}\frac{A_{s}}{A_{c}}\tau_{b1}x\frac{1}{\frac{\sigma_{s,\max}}{E_{s}}-\frac{4}{\Phi_{s}E_{s}}\tau_{b1}x} & for & x<\frac{\sigma_{s,\max}}{E_{s}}\frac{\Phi_{s}E_{s}}{4\tau_{b1}}\\ E_{ct,\lim} & for & x\geq \frac{\sigma_{s,\max}}{E_{s}}\frac{\Phi_{s}E_{s}}{4\tau_{b1}} \end{array}.$$

**Figure 6:**
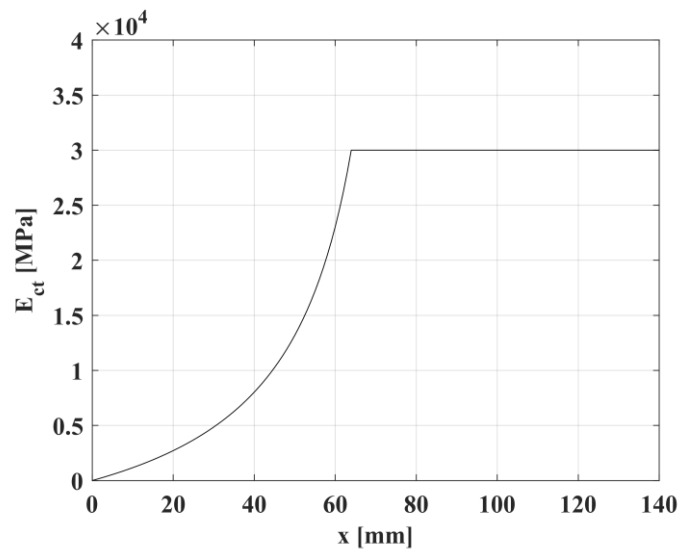
Trend of the elasticity modulus in tension, E_ct_, as a function of the crack distance assumed within this work.

In Figure 6, the trend of the elasticity modulus in tension, E_ct_, evaluated by adopting Equation (9) is shown.

## 3. Flexural Behavior of a Circular Section Considering Tension Stiffening

**Figure 7:**
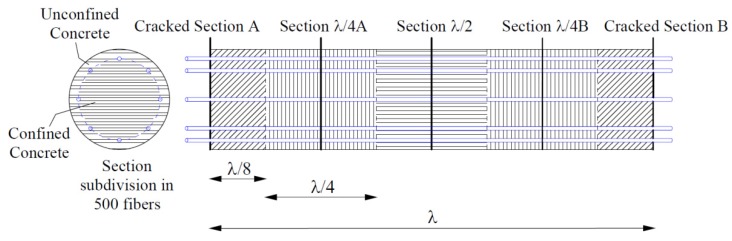
Element portion comprised between two consecutive cracks: (**a**) Fibers of the circular section; (**b**) sections studied for the evaluation of the total rotation.

The moment–rotation behavior of the circular cross section portion of an element that is comprised between two consecutive cracks can be obtained by integrating the moment–curvature relationship along the length of the element itself, and adopting for the concrete in tension the fictitious elasticity modulus given by Equations (6), (7), and (9). Alternatively, as assumed in the present study, the element can be discretized in smaller elements, and the curvature considered to be constant within each element. The rotation, Θ_A-B_, between the two consecutive cracked sections can be so evaluated as follows:

(10) $$\Theta_{A-B}=\chi_{A}\frac{\lambda }{8}+\chi_{\frac{\lambda }{4}A}\frac{\lambda }{4}+\chi_{\frac{\lambda }{2}}\frac{\lambda }{4}+\chi_{\frac{\lambda }{4}B}\frac{\lambda }{4}+\chi_{B}\frac{\lambda }{8}$$

where χ_A_, χ_λ/4A_, χ_λ/2_, χ_λ/2B_, and χ_B_ are, with reference to Figure 7, the curvature evaluated respectively at the cracked section A, at a distance equal to λ/4 from section A, in the middle between the two consecutive cracked sections A and B, at a distance equal to λ/4 from section A, and at the cracked section B.

To evaluate the moment–curvature relationship, each section was so discretized into a finite number of longitudinal fibers (Figure 7), distinguishing the confined and unconfined concrete zones in compression and the concrete part influenced by the tension stiffening. The hypotheses of plane sections and absence of slip between steel bars and the surrounding concrete were adopted. The effective area of concrete in tension was obtained following the Eurocode 2 [1] approach.

**Figure 8:**
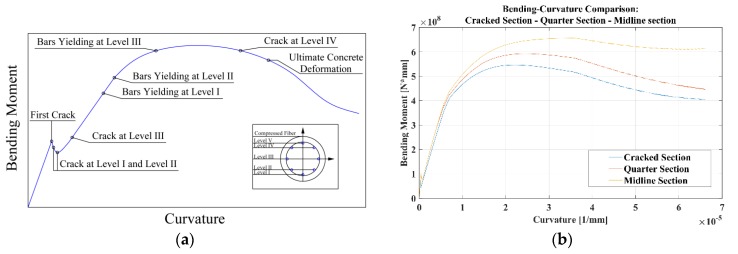
Examples of the moment-curvature curve computed for the circular pile studied by Teherani [16]: (**a**) typical shape for the cracked section; (**b**) comparison between the computed behaviors at cracked, λ/4, and λ/2 sections.

The concrete behavior in compression was modeled using the Mander [24] approach for the confined zone and the Popovics [25] one for the unconfined concrete. For the concrete in tension a brittle linear elastic behavior, as proposed by CEB-fib Model Code 2010 [2], was used. A bilinear hardening behavior was assumed for the steel reinforcing bars. An example of the resulting moment-curvature curve, evaluated in correspondence of a cracked section, is shown in Figure 8a. The effects of tension stiffening are evident when the moment-curvature curve, evaluated on the cracked section at λ/4 and at λ/2, is compared (Figure 8b). It can be seen that, thanks to the presence of concrete in tension between the two cracks, the ultimate bending moment of the λ/4 and λ/2 sections is greater than the cracked section’s one, meaning that the plastic rotation of the element tends to accumulate within the cracked sections.

The displacement of a circular cross section element in bending was then easily estimated, by subdividing it into blocks. The length of each block in the cracked portion of the element was assumed to be equal to the crack’s distance. In the uncracked portion it was assumed to be the maximum block length observed in the cracked zone.

**Figure 9:**
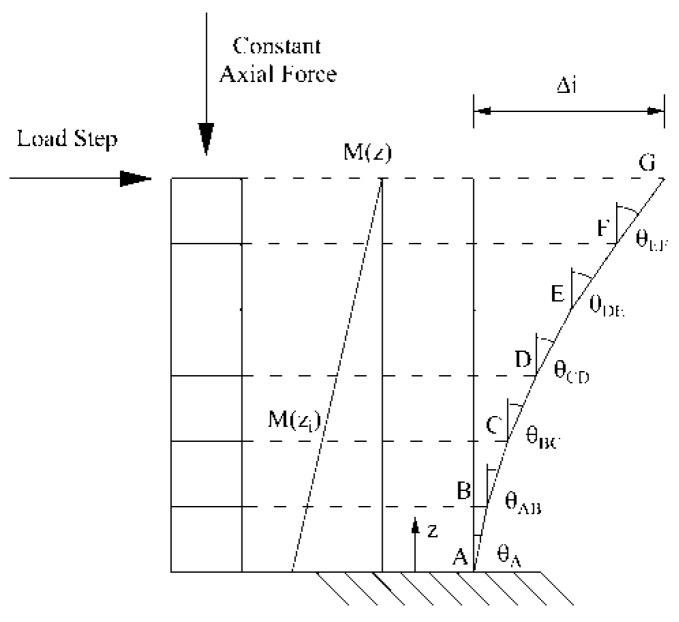
Evaluation of the relative displacement between two sections of an element in bending.

The relative displacement between two sections, Δ_tot_, was computed by summing the displacements, δ_i_, of each block comprised between the two sections.

(11) $$\Delta_{tot}=\sum_{i=1}^{N}\delta_{i}=\sum_{i=1}^{N}\left(H_{i}\sum_{j=1}^{i}\Theta_{j}\right)$$

where H_i_, is the length of the i block; N is the number of blocks in which the portion of element is divided into; and Θ_i_ is the relative rotation between the two faces of the i block evaluated using Equation (10), as shown in Figure 9.

## 4. Experimental Validation

The procedure described in the previous paragraphs was validated by applying it to evaluate the load-deflection curves of several r.c. circular cross section elements, and then comparing them with the experimental results.

### 4.1. Description of the Experimental Results

**Figure 10:**
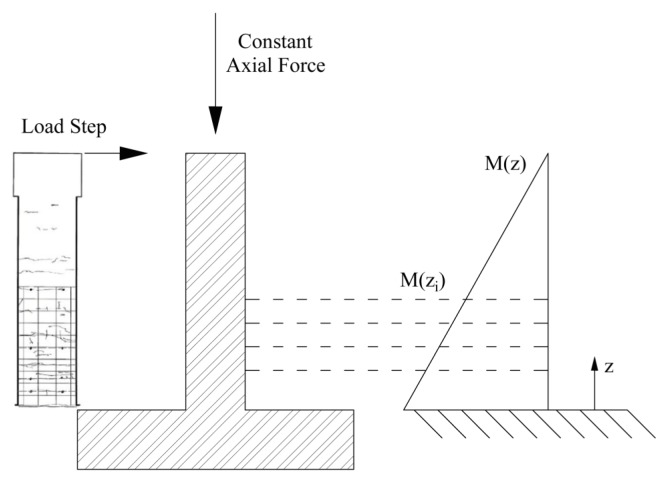
Bending Moment Profile $M\left(z_{i}\right)$.

For this purpose, the experimental tests conducted by Lehman and Moehle [26] and by Calderone and Lehman [27] were used. Both publications provide a lot of details regarding the geometrical and mechanical characteristics of the section, on the loading mode, and the cracks pattern during the test and on its completion. In both cases the test setup was equal: the column is fixed to the base and loaded transversely by a cyclic force and axially by a constant one, as shown in Figure 10.

Lehman and Moehle [26] tested a total of five specimens, while focusing the study on the influence of the slenderness ratio on column behavior. Table 1 summarizes the main geometrical and mechanical characteristics of the specimens. All of the the columns were subjected, during the experiments, to an external compressive force equal to 654 kN. Calderone and Lehman [27] tested four specimens, while varying the column slenderness and the transversal reinforcement as shown in Table 2. All of the columns were subjected to an external compressive force equal to 720 kN.

In both cases, the transverse load was applied cyclically, and with an increasing amplitude. The envelope curve of each test was taken into consideration in order to compare the monotonic experimental behavior with the numerical behaviour. It was also assumed that phenomena such as low cycle fatigue do not influence the response of the column, given the low values of the imposed displacements (the maximum value of interest is around 5 cm).

### 4.2. Comparison of Numerical and Experimental Results

The cracks pattern recorded by Lehman and Moehle [26] and Calderone and Lehman [27] allowed a preliminary validation of the mean crack distance value given by Equation (11). Table 3 shows the comparison between the numerical and experimental results. Considering that the identification of “cracks” is characterized by high uncertainty, and that their actual interaxis is strongly influenced by the actual mechanical characteristics of the component materials, Table 3 shows a good mean agreement between the numerical and experimental results.

**Figure 11:**
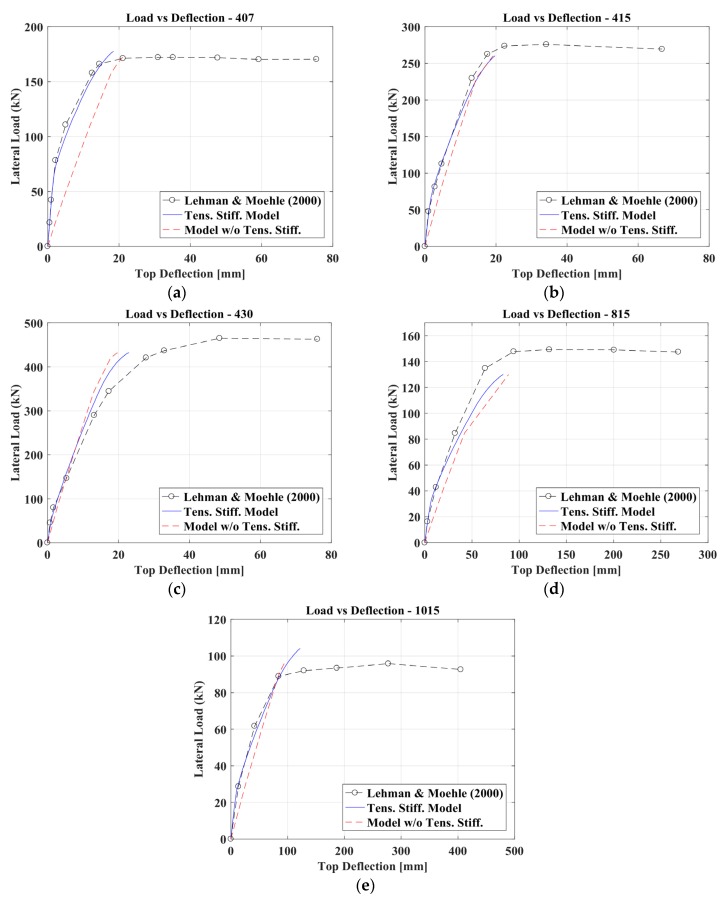
Comparison between the experimental results of Lehman and Moehle [26] and the numerical ones in terms of initial stiffness: Column IDs (**a**) 407, (**b**) 415, (**c**) 430, (**d**) 815 and (**e**) 1015.

**Figure 12:**
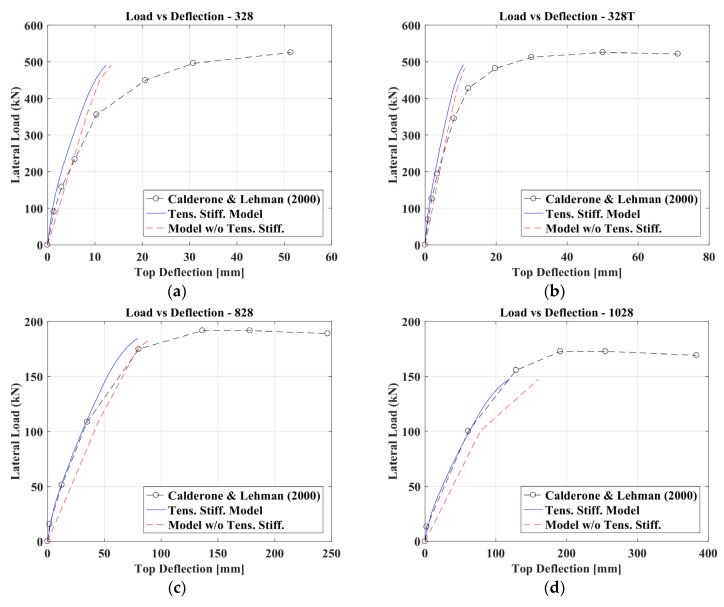
Comparison between the experimental results of Calderone and Lehman [27] and the numerical ones in terms of initial stiffness: Column IDs (**a**) 328, (**b**) 328T, (**c**) 828 and (**d**) 1028.

Using the bending moment profile M(z) along the column calculated at each loading step, the corresponding deflections were obtained by adopting the procedure described in the previous paragraphs. The numerical curves were evaluated up to the point corresponding to the peak stress in the concrete in compression. The focus of this research is, indeed, to evaluate the influence of tension stiffening on flexural stiffness. So, the behavior of the r.c. elements with circular sections beyond this point was not investigated within this research. The comparison between the experimental and numerical results, as shown in Figure 11 and Figure 12, testifies to the optimal capacity of the proposed model in evaluating the flexural stiffness of the circular cross section columns. The figures show also the equivalent force-displacement curve obtained, not considering the contribution of the concrete in tension. For the comparison among the proposed method’s curves (considering also tension stiffening effects), the numerical curve obtained—ignoring tension stiffening and the experimental results—showed that:

− for column 407, not considering tension stiffening can lead to a sensible error in evaluating the element’s stiffness;
− for more slender columns (828 and 1028), tension stiffening effects are more evident than for shorter ones (328 and 328T).

## 5. Parametric Analysis

To study the influence of tension stiffening on global behavior, in this section a parametric analysis was performed for different cross sections, varying: the diameter, the longitudinal reinforcing ratio, and the compressive force. The cases considered in the parametric analysis are summarized in Table 4. Three different diameters, with values typical of pile foundations (60 cm, 100 cm, and 150 cm), and three longitudinal reinforcing bar ratios (1%, 2%, and 3%) were considered. For each of these sections, the influence of four levels of external compressive force was studied. These force levels correspond to 5%, 10%, 25%, and 35% of the ultimate axial resistance of the section (Nu), evaluated with the expression: $N_{u}=f_{c}A_{c}$.

The results presented herein were obtained in two ways: one by taking into account the influence of the tension stiffening, and adopting the model proposed in the previous paragraphs; the other by neglecting it. It was so possible to point out how tension stiffening can affect the performance of a reinforced concrete element with a circular section.

Assuming that the concrete elastic modulus remains constant, the influence of tension stiffening was evaluated in terms of an equivalent moment of inertia. For every diameter considered and listed in Table 4, two plots were presented, the first referring to a normalized moment of inertia defined as the ratio between the secant flexural rigidity (EI)_sec_ at the first steel bar yielding and the intact flexural rigidity of the section (E_c_I = E_c_πD^4^/64), and the second referring to a normalized moment of inertia defined as the ratio between the secant flexural rigidity evaluated when the concrete reaches peak stress in compression and the intact flexural stiffness.

**Figure 13:**
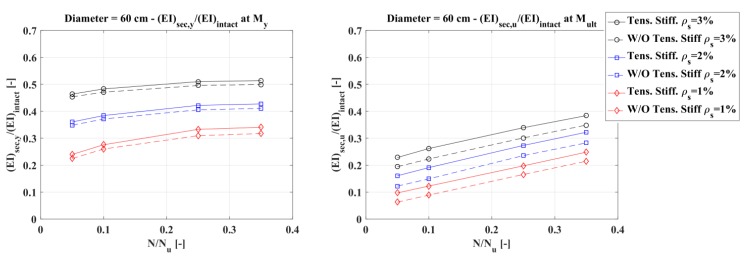
Diameter 60 cm: normalized secant stiffness at first steel bar yielding (**left**) and at peak stress in compression (**right**).

**Figure 14:**
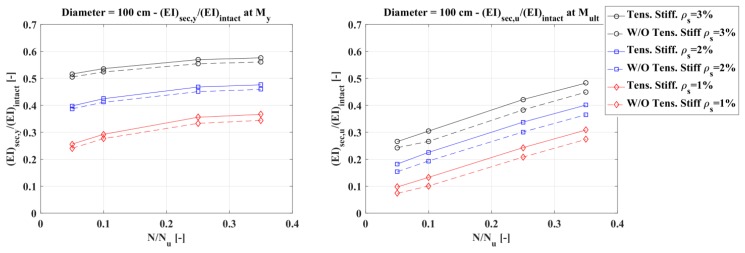
Diameter 100 cm: normalized secant stiffness at first steel bar yielding (**left**) and at peak stress in compression (**right**).

**Figure 15:**
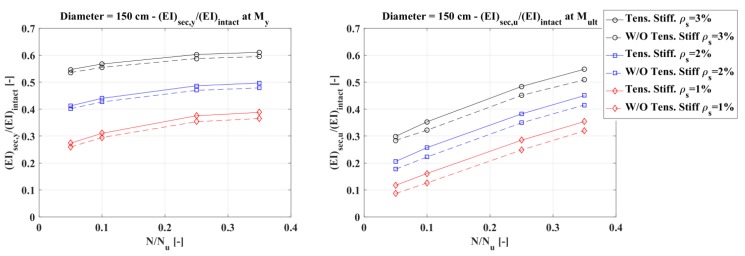
Diameter 150 cm: normalized secant stiffness at first steel bar yielding (**left**) and at peak stress in compression (**right**).

Figure 13, Figure 14 and Figure 15 show that the influence of tension stiffening is:

− less evident on the secant stiffness at the first bar yielding than on that evaluated at the maximum bending moment;
− increasing as the reinforcement ratio decreases;
− not so much influenced by the axial force, except for low values of the reinforcement ratio; and
− higher for smaller diameters.

## 6. Conclusions

Within this paper, the influence of tension stiffening on the global behavior of elements with a circular cross section and subjected to the combined axial–flexural action was studied. A preliminary analysis of the modeling approach adopted by Salvatore et al. [8] was carried out; it was then slightly modified in order to avoid problems in the formal definition of the elasticity modulus in tension E_ct_.

The comparison of numerical results, obtained with the enhanced model, and the experimental ones, provided by the studies of Lehman and Moehle [26] and Calderone and Lehman [27], highlighted the capacity of the model to foresee the flexural behavior of r.c. elements characterized by a circular section, especially from a stiffness point of view. A lack of precision was noted in the evaluation of the flexural resistance, but this aspect is practically independent from the tension stiffening phenomenon, and it can be mainly ascribed to the lack of information about the actual mechanical behaviour of the constituent materials. The analysis of the results also highlighted the importance of tension stiffening in the evaluation of flexural stiffness, especially for slender columns.

The parametric analysis carried out using the enhanced model, and varying the values of the axial force and reinforcement ratio, highlighted that the influence of tension stiffening:

− is less evident on the yield stiffness than that of the one evaluated at the moment corresponding to the reaching of peak stress in compression in concrete;
− increases as the reinforcement ratio decreases;
− is not so much influenced by the axial force, except for low values of the reinforcement ratio; and
− is higher for smaller section diameters.

These results suggests that for r.c. elements characterized by a circular section with a diameter larger than 1 m and with a reinforcement ratio higher than 1%, such as usual bridge piers, the influence of tension stiffening can be neglected.

The influence of tension stiffening becomes sensible for low diameters (around 60 cm, such as some foundation piles) and low values of the reinforcement ratio (lower than 1%). However, in general, in the case of foundation piles, it can be easily assumed that the influence of tension stiffening on global behavior is absorbed by the uncertainties in the definition of the soil’s mechanical properties.

## Figures and Tables

**Table 1:** Main geometrical and mechanical properties of specimens tested by Lehman and Moehle [26].

Column ID	Height (mm)	Section Diameter (mm)	Concrete Cover (mm)	Long. Reinforcement [Number and Diameter (mm) of Bars]	Transverse Reinforcement [Diameter (mm)/Spacing (mm)]	Concrete *f’c* (MPa)	Long. Reinforcement *f_y_* (MPa)	Trans. Reinforcement *f_y_* (MPa)
407	2438	609.6	33.4	11 Φ16	Φ6/32	43.4	471.6	668.1
415	2438	609.6	33.4	22 Φ16	Φ6/32	43.4	471.6	668.1
430	2438	609.6	33.4	44 Φ16	Φ6/32	43.4	471.6	668.1
815	4877	609.6	33.4	22 Φ16	Φ6/32	43.4	471.6	668.1
1015	6096	609.6	33.4	22 Φ16	Φ6/32	43.4	471.6	668.1

**Table 2:** Main geometrical and mechanical properties of specimens tested by Calderone and Lehman [27].

Column ID	Height (mm)	Section Diameter (mm)	Concrete Cover (mm)	Long. Reinforcement [Number and Diameter (mm) of Bars]	Transverse Reinforcement [Diameter (mm)/Spacing (mm)]	Concrete *f’c* (MPa)	Long. Reinforcement *f_y_* (MPa)	Trans. Reinforcement *f_y_* (MPa)
328	1829	609.6	41.3	28 Φ19	Φ6/25	27.6	483.0	483.0
328T	1829	609.6	41.3	28 Φ19	Φ6/76	27.6	483.0	483.0
828	4877	609.6	41.3	28 Φ19	Φ6/76	27.6	483.0	483.0
1028	6096	609.6	41.3	28 Φ19	Φ6/51	27.6	483.0	483.0

**Table 3:** Comparison between the experimental and numerical values of the mean crack distance.

Column ID	Experimental Mean Crack Distance * (mm)	Mean Cracks Distance Evaluated by Equation (12) (mm)	Column ID	Experimental Mean Crack Distance * (mm)	Mean Cracks Distance Evaluated by Equation (12) (mm)
407	167	204	328	78	74
415	93	112	328T	77	74
430	114	79	828	114	74
815	147	112	1028	84	74
1015	102	112	-	-	-

* Evaluated in correspondence of the first meter starting from the column base.

**Table 4:** Circular reinforced concrete sections used for the parametric study.

Diameter (m)	Longitudinal Bars	As/Ac	N/Nu (1)	N/Nu (2)	N/Nu (3)	N/Nu (4)
0.60	14 ϕ16	1%	0.05	0.10	0.25	0.35
0.60	18 ϕ20	2%	0.05	0.10	0.25	0.35
0.60	22 ϕ22	3%	0.05	0.10	0.25	0.35
1.00	30 ϕ18	1%	0.05	0.10	0.25	0.35
1.00	42 ϕ22	2%	0.05	0.10	0.25	0.35
1.00	44 ϕ26	3%	0.05	0.10	0.25	0.35
1.50	40 ϕ24	1%	0.05	0.10	0.25	0.35
1.50	44 ϕ32	2%	0.05	0.10	0.25	0.35
1.50	66 ϕ32	3%	0.05	0.10	0.25	0.35
